# Adrenal insufficiency is common amongst kidney transplant recipients receiving maintenance prednisolone and can be predicted using morning cortisol

**DOI:** 10.1093/ndt/gfac044

**Published:** 2022-05-13

**Authors:** Maria Tomkins, Julie Martin-Grace, Carmel Kennedy, Olive McEnroe, Karen Heverin, Shari Srinivasan, Dilly Little, Peter Conlon, Declan De Freitas, Mark Denton, Colm Magee, Conall O'Seaghdha, Michael W O'Reilly, Chris J Thompson, Mark Sherlock

**Affiliations:** Department of Endocrinology, Beaumont Hospital, Dublin, Ireland; Department of Endocrinology, Royal College of Surgeons in Ireland, Dublin, Ireland; Department of Endocrinology, Beaumont Hospital, Dublin, Ireland; Department of Endocrinology, Royal College of Surgeons in Ireland, Dublin, Ireland; Department of Endocrinology, Beaumont Hospital, Dublin, Ireland; Transplant Urology Nephrology Directorate, Beaumont Hospital and Royal College of Surgeons in Ireland, Dublin, Ireland; Department of Clinical Biochemistry, Beaumont Hospital, Dublin, Ireland; Department of Clinical Biochemistry, Beaumont Hospital, Dublin, Ireland; Transplant Urology Nephrology Directorate, Beaumont Hospital and Royal College of Surgeons in Ireland, Dublin, Ireland; Transplant Urology Nephrology Directorate, Beaumont Hospital and Royal College of Surgeons in Ireland, Dublin, Ireland; Transplant Urology Nephrology Directorate, Beaumont Hospital and Royal College of Surgeons in Ireland, Dublin, Ireland; Transplant Urology Nephrology Directorate, Beaumont Hospital and Royal College of Surgeons in Ireland, Dublin, Ireland; Transplant Urology Nephrology Directorate, Beaumont Hospital and Royal College of Surgeons in Ireland, Dublin, Ireland; Transplant Urology Nephrology Directorate, Beaumont Hospital and Royal College of Surgeons in Ireland, Dublin, Ireland; Department of Endocrinology, Beaumont Hospital, Dublin, Ireland; Department of Endocrinology, Royal College of Surgeons in Ireland, Dublin, Ireland; Department of Endocrinology, Beaumont Hospital, Dublin, Ireland; Department of Endocrinology, Royal College of Surgeons in Ireland, Dublin, Ireland; Department of Endocrinology, Beaumont Hospital, Dublin, Ireland; Department of Endocrinology, Royal College of Surgeons in Ireland, Dublin, Ireland

**Keywords:** adrenal insufficiency, cortisol, kidney transplant, prednisolone, synacthen

## Abstract

**Background:**

Long-term glucocorticoid therapy is a key component of immunosuppression for kidney transplant recipients (KTRs), leading to significant cumulative glucocorticoid exposure. The aims of this study are to investigate the prevalence of adrenal insufficiency (AI) in KTRs taking prednisolone and to develop a screening algorithm to identify patients at the highest risk of AI.

**Methods:**

In this cross-sectional cohort study, 67 KTRs receiving prednisolone underwent a short synacthen test (SST) and measurement of cumulative glucocorticoid exposure.

**Results:**

A total of 72% (*n* = 48) of participants failed the SST. Participants with AI had a higher daily prednisolone dose (4.9 versus 4.2 mg/day; *P* = .002) and greater cumulative glucocorticoid exposure (289 versus 111 mg/kg; *P* = .03) than those with intact adrenal function. Participants with AI had lower baseline cortisol than participants with intact adrenal function (143 versus 303 nmol/L; *P* < .001). Morning cortisol of >288 nmol/L predicted a normal SST with 100% specificity [95% confidence interval (CI) 92–100] and 70% sensitivity (95% CI 56–78%), therefore excluding AI.

**Conclusions:**

Our results suggest KTRs are at a higher risk for AI than previously reported. A morning serum cortisol measurement is a useful screening tool in this cohort, reducing the need for stimulatory testing by 44%. KTRs with AI need education regarding glucocorticoid sick rules, similar to patients with other forms of AI.

## Graphical Abstract

**Graphical Abstract:**
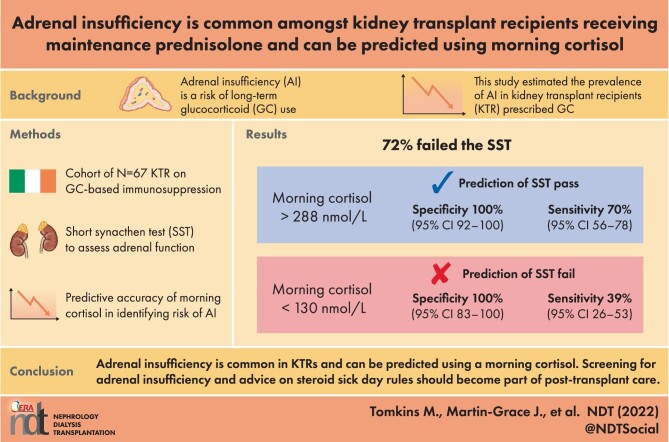

KEY LEARNING POINTS **What is already known about this subject?**

- Meta-analysis data suggest that the absolute risk of adrenal insufficiency (AI) in kidney transplant recipients (KTRs) is 56.2; however, this is based on previous studies performed in an era of higher maintenance prednisolone doses, with varying methods to assess adrenal function.
- Currently there are no guidelines for the appropriate screening, monitoring and management of AI in the KTR population.

**What this study adds?**

- This is the largest cross-sectional study to report the risk of AI in KTRs receiving low-dose glucocorticoid-based immunosuppression and is one of the few adult studies to examine the effect of cumulative glucocorticoid exposure on the risk of AI. It is the only such study to include pretransplant glucocorticoid exposure.
- This study suggests that AI is more common than previously reported in KTRs receiving low-dose prednisolone therapy.
- A basal serum cortisol concentration is a useful screening tool for identifying KTRs at risk of AI.

**What impact this may have on practice or policy?**

- Clinicians should have a high clinical suspicion of adrenal crisis in this patient group and treat suspected adrenal crisis without delaying confirmatory testing.
- We would recommend routine screening for AI in KTRs prescribed glucocorticoid immunosuppression. We suggest that KTRs with a morning cortisol concentration <288 nmol/L who are receiving ≥5 mg prednisolone/day should be considered at high risk of AI and should follow steroid sick day rules.
- Sick day rule education should become part of the routine care for KTRs prescribed glucocorticoid immunosuppression.

## INTRODUCTION

Adrenal insufficiency (AI) occurring after the suppression of the hypothalamic–pituitary–adrenal axis by exogenous glucocorticoids is termed tertiary adrenal insufficiency (TAI) [1]. TAI is associated with all forms of exogenous glucocorticoid medication and patients exposed to glucocorticoids for prolonged periods are at an increased risk of TAI [2]. Patients with undiagnosed TAI are at risk of developing a life-threatening adrenal crisis should they discontinue steroid therapy abruptly or if they encounter a physiological stressor such as an intercurrent illness or surgery without appropriate stress dose steroid dosing [3, 4].

Glucocorticoid medications such as prednisolone are commonly used as a component of long-term maintenance immunosuppression after kidney transplantation, and high-dose glucocorticoids are the first-line treatment for acute rejection episodes [5, 6]. Current transplant guidelines recommend daily doses of prednisolone of ≤5 mg [5, 6]. As the median survival following a kidney transplant is excellent [7], many kidney transplant recipients (KTRs) receive a significant cumulative dose exposure of glucocorticoids over the lifetime of the graft and consequently are at risk of developing AI and associated adrenal crises. However, despite this, steroid safety advice and sick day rule education do not feature in the major clinical practice guidelines for the management of KTRs [5, 6].

A small number of studies have reported a 44–80% prevalence of TAI in the KTRs population [8–11] (Table 1); meta-analysis data suggest that the absolute risk of TAI in this population is 56.2 [(95% confidence interval (CI) 42.9–68.6] [2]. However, most published studies have small participant numbers [8–16], were performed in an era of higher maintenance prednisolone doses (5–20 mg prednisolone/day) and vary in their methods of adrenal function assessment [10, 13-15]. More recent studies of patients treated with lower maintenance prednisolone (5–7.5 mg prednisolone/day) suggest the prevalence is 31–60% [14–16]. It is therefore difficult to predict which KTR patients are likely to develop TAI on modern glucocorticoid regimens. While there is some evidence in the paediatric population that prescribed daily dose correlates with risk of TAI [15], the duration of glucocorticoid exposure has not consistently correlated with the risk of TAI [14, 16] and there is a lack of published data detailing how cumulative glucocorticoid exposure correlates with the risk of TAI.

**Table 1.** Summary of existing evidence base for TAI in KTRs

Reference	Cohort	Steroid dose at enrolment (prednisone/day)	AI (%)	Assessment	Comment
Naik *et al.* [9]	KTR (*n* = 10)	10 mg	80	SST	SST on participants ≤5mg, 4/5 fail, 1 participant symptomatic AI
Canaflax *et al.* [10]	KTR (*n* = 18)	0.1–1 mg/kg/day	44	250 µg SST	50% of SST failures died within 2 years, 12 months pred >25 g cumulative dose predicts SST failure
Rodgers *et al.* [11]	KTR (*n* = 21)	15 mg/day (25 g)	66	SST ITT	Pass SST confirmed using ITT
Shapiro *et al.* [8]	KTR (*n* = 13)	5–20 mg	46	SST	
Bromberg *et al.* [12]	KTR (*n* = 40)	5–10 mg	63	SST	Admission with stress events, no stress dose given, 5/40 hyponatraemia, 7/40 hypotension
Boots *et al.* [45]	KTR (*n* = 53)	10 mg	44	SST	After 6 months of prednisolone exposure (33% failed SST after 3 months)
Miozzari *et al.* [13]	KTR (*n* = 63 EMC and 32 SST)	10 mg	45 (31 SST, 14 EMC)	EMC 1 µg SST	EMC measured prior to withdrawal steroids 14% low EMC Subgroup SST pre-/post-withdrawal (*n* = 32) 31% failed SST 31% symptoms of AI on steroid withdrawal
Baz-Hecht *et al.* [14]	KTR (*n* = 37) SPK (*n* = 11)	5 mg	60	1 µg SST	SST repeated, 3/12 off steroids in 13 participants 5/13 passed repeat SST
Bilavsky *et al.* [15]	pKTR (*n* = 29) pLTR (*n* = 19)	≤0.5 mg/kg/day	31.2 (steroid group)	250 µg SST	28/29 KTR on steroid immunosuppression 31.2% AI (versus 6.2% AI in control group)
Valentin *et al.* [16]	KTR (*n* = 30) RRT (*n* = 30) (*n* = 60)	5–7.5 mg	43	250 µg SST	43% KTRs failed SST versus 3.2% RRT control group 7.5 mg/day: 80% failed SST 5 mg/day: 36% failed SST

ITT, insulin tolerance test; SPK, simultaneous pancreas kidney transplant; RRT, renal replacement therapy; LTR, liver transplant recipient; pLTR, paediatric liver transplant recipient; EMC, early morning cortisol; pred, prednisolone.

TAI can be asymptomatic outside of the setting of an adrenal crisis [2] and therefore clinicians rely heavily on laboratory investigations for diagnosis. Stimulatory tests such as the short synacthen test (SST) and insulin tolerance test are the gold standard for the diagnosis of AI [17, 18] and are safe and well-tolerated investigations. The use of a morning serum cortisol value as a screening tool has been reported in other patient cohorts to reduce the need for stimulatory testing by 40% [19–21]. This would represent time- and resource-saving benefits for both patients and healthcare systems; however, it has not yet been validated in this patient cohort.

We hypothesize that TAI is highly prevalent in the KTR patient population and that it is underdiagnosed and therefore undertreated. We suggest that the risk of TAI correlates with both daily prednisolone dose and cumulative glucocorticoid exposure.

The primary aim of this study is to determine the prevalence of TAI in renal transplant recipients receiving maintenance immunosuppression ≤5 mg prednisolone/day. Secondary aims were to assess the relationship between TAI and cumulative dose exposure to prednisolone, to evaluate the use of a morning serum cortisol concentration as a screening tool for the diagnosis of TAI in this group and to assess knowledge of steroid sick day rules in the KTR population.

## MATERIALS AND METHODS

### Participant recruitment

Participants were recruited consecutively from the National Kidney Transplant outpatient department at Beaumont Hospital, Dublin, Ireland. Eligible patients were KTRs $\ge $18 years of age, ≥1 year post-transplant, established on a stable maintenance dose of oral prednisolone for ≥6 months, with stable graft function [estimated glomerular filtration rate (eGFR) >30 mL/min/1.73 m^2^]. Exclusion criteria included known hypothalamic–pituitary or adrenal pathology, known adrenal insufficiency, critically ill patients, pregnancy or oral oestrogen medication within 6 weeks of study enrolment. Participants with impaired graft function (eGFR <30 mL/min/1.73 m^2^) were excluded from the study due to changes in endogenous cortisol metabolism and binding globulin affinity and changes in prednisolone pharmacokinetics in the latter stages of chronic kidney disease [22–24].

### Study visit

Patients attended for a single visit, during which a detailed medical history was obtained and electronic and paper healthcare records were reviewed. A detailed cumulative glucocorticoid exposure history was calculated, which included pretransplant, perioperative and post-transplant exposure to glucocorticoids and exposure to glucocorticoid medication for reasons unrelated to transplantation (such as the treatment of underlying inflammatory conditions), using patient interview and hospital chart review. The results were finalized after review of the National Kidney Disease Clinical Patient Management System, a national kidney disease electronic patient management system that is used in the care of patients with chronic kidney disease. Knowledge of steroid sick day rules advice, medical alert card and emergency hydrocortisone administration was assessed by participant interview.

Participants were requested to withhold their morning medications, as per local practice, to obtain trough drug levels. Fasting laboratory investigations were performed, including full blood count; renal, liver and bone profiles; total serum bicarbonate, magnesium, serum albumin, tacrolimus or sirolimus levels as appropriate and plasma adrenocorticotropin (ACTH), using in-hospital assays (see below for details). Cortisol binding globulin concentration was measured using a competitive radioimmunoassay (DIAsource Immunoassays, Louvain-la-Neuve, Belgium) [intra-assay coefficient of variation (CV) 8.6% at 23 µg/mL and 3.9% at 83 µg/mL].

Blood samples were taken prior to (0 minutes) and 30 minutes after 250 µg intravenous bolus of synthetic ACTH was administered (Synacthen, Alfa Sigma, Rome, Italy). Serum cortisol concentration was measured using the Elecsys II Cortisol immunoassay (Roche Diagnostics, Mannheim, Germany), with a CV of 1.8–2.1% and cross-reactivity with prednisolone of 7.9%. All laboratory samples were obtained before 10 a.m. A peak cortisol response to SST >430 nmol/L was regarded as a normal response [25, 26]. Participants who did not achieve a peak cortisol response of >430 nmol/L were educated regarding sick day steroid rules, in accordance with Society for Endocrinology recommendations [4]. Baseline ACTH concentration was analysed using the Elecsys ACTH immunoassay (Roche Diagnostics).

The study was reviewed and approved by the Beaumont Hospital Research Ethics Committee (reference 19/72). Written informed consent was obtained from each participant prior to enrolment in the study and the study was conducted in accordance with the ethical standards laid down in the 1964 Declaration of Helsinki.

### Statistical analysis

Data are expressed as number (%) for categorical variables and as median [interquartile range (IQR)] for non-parametric continuous variables. The *t*-test was used to compare single variables and the Mann–Whitney test was used to determine the statistical significance of the analysis of multiple comparisons of non-parametric variables. Fisher’s exact test was used to analyse the association between categorical variables. A receiver operating characteristics (ROC) analysis was performed to assess basal cortisol as a predictor of SST response, with an area under the curve (AUC) of 1.0 representing perfect discrimination. A *P*-value <.05 was considered statistically significant. Statistical analysis was performed using the GraphPad Prism software package (GraphPad Software, San Diego, CA, USA).

## RESULTS

### Cohort description

**FIGURE 1:**
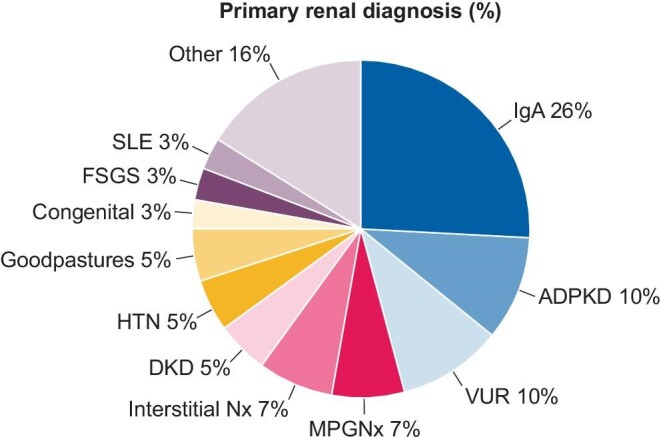
Primary renal diagnoses of study participants. IgA, immunoglobulin A nephropathy; ADPKD, autosomal dominant polycystic kidney disease; VUR, vesicoureteric reflux; MPGNx, membranoproliferative glomerulonephritis; interstitial Nx, interstitial nephritis; DKD, diabetic kidney disease; HTN, hypertensive kidney disease; FSGS, focal segmental glomerulosclerosis; SLE, systemic lupus erythematosus.

A total of 67 participants were recruited between December 2019 and November 2020, 64% of whom were male (Table 2). The median age of participants was 52 years (IQR 39–64) and the median duration since transplant was 79 months (IQR 46–146). The median duration of dialysis prior to transplantation was 24 months (IQR 11–42). The most common transplant type was a deceased donor (72%) (Table 2). Twenty-one percent of participants had more than one previous renal transplant. Most participants were also taking a calcineurin inhibitor (81%) and mycophenolate mofetil (82%) as immunosuppressive therapy, with a median tacrolimus level at the time of study visit of 6.6 ng/mL (IQR 5.6–7.8). The primary renal diagnosis in the study cohort was reflective of that of the Irish national KTR population (Figure 1).

**Table 2.** Participant characteristics

Characteristics	All participants	Pass	Fail	*P*-value (pass versus fail)
Demographics				
Patients, *n* (%)	67	19 (28)	48 (72)	
Age (years), median (IQR)	52 (39–64)	45 (35–56)	55 (35–56)	.095
Male, *n* (%) Female, *n* (%)	43 (64) 24 (36)	16 (84) 3 (16)	27 (56) 21 (44)	.047
BMI, median (IQR)	26 (23–30)	26 (24–30)	27 (23–30)	.96
eGFR (mL/min/1.73 m^2^), median (IQR)	69 (52–84)	81 (60–98)	63 (52–83)	.03
Transplant history				
Previous transplant, *n* (%)	14 (21)	2 (11)	12 (25)	.3
Transplant type, *n* (%) DCD LRD LURD SPK	48 (72) 14 (21) 4 (6) 1 (1)	14 (74) 2 (11) 2 (11) 1 (5)	34 (71) 12 (25) 2 (4) 0	
Time since transplant (months), median (IQR)	79 (46–146)	77 (39–146)	84 (47–150)	.98
Time since first transplant (months), median (IQR)	99 (49–254)	77 (39–146)	103 (53–268)	.23
Tacrolimus use, *n* (%)	54 (81)	17 (89)	37 (77)	.32
Mycophenolate mofetil use, *n* (%)	55 (82)	17 (89)	38 (79)	.49
Tacrolimus dose (mg/kg/day), median (IQR)	0.043 (0.03–0.06)	0.047 (0.04–0.06)	0.038 (0.026–0.06)	.3
Tacrolimus level (ng/mL), median (IQR)	6.6 (5.6–7.8)	6.2 (5.4–7.7)	6.6 (5.8–7.8)	.38

BMI, body mass index; DCD, deceased donor; LRD, living related donor; LURD, living unrelated donor; SPK, simultaneous pancreatic–kidney transplant.

### Prevalence of AI

A total of 48 participants (72%) failed an SST, indicating AI. Only 19 participants had a normal response, indicating preserved adrenal function. Participants with AI had a lower eGFR than those with intact adrenal function [63 mL/min/1.73 m^2^ (IQR 52–8) versus 81 (60–98); *P* = .03], but there was no significant difference in age, body mass index, duration since transplantation, history of a previous transplant, use of calcineurin inhibitors or mycophenolate mofetil, daily tacrolimus dose (mg/kg), pretransplant glucocorticoid exposure or exposure to other glucocorticoid preparations between the two groups.

### Glucocorticoid exposure

Cumulative glucocorticoid exposure was associated with a risk of AI. Participants with AI had a higher median daily dose of prednisolone [4.9 mg/day (IQR 4.7–5) versus 4.2 (3.6–4.8); *P* = .002], a greater absolute cumulative glucocorticoid exposure [14 775 mg prednisolone (IQR 8193–37 275) versus 9220 (5450–21 850); *P* = .056] and higher weight-based cumulative exposure [289 mg prednisolone/kg (IQR 115–489) versus 111 (72–284); *P* = .03] than participants with preserved adrenal function. AI was observed in patients receiving both daily and alternate-day prednisolone regimes and in participants receiving maintenance doses as low as 2.5 mg prednisolone/day.

### Short synacthen testing

Participants who passed an SST had a significantly higher median basal cortisol concentration [303 nmol/L (IQR 238–342) versus 143 (90–211); *P* < .001], higher stimulated cortisol [490 nmol/L (IQR 469–531) versus 325 (272–375); *P* < .001] and greater delta cortisol in response to synacthen stimulation [187 nmol/L (IQR 151–272) versus 139 (102–195); *P* = .013] than the AI group (Table 3, Figure 3). Basal ACTH concentration was suppressed in the AI group [13.5 pg/mL (IQR 7.3–18.9) versus 20 (12.6–23.6); *P* = .016], reflecting hypothalamic–pituitary–adrenal axis suppression. While there was a statistically significant difference in serum albumin concentration, the median concentration values fell within the reference range in both groups [42 g/L (IQR 40–45) versus 45 (41–47), reference interval 35–52 g/L] and there was no significant difference in cortisol binding globulin concentration between the two groups [51.1 mg/L (IQR 44.2–59.1) versus 54.4 (50.8–62.5); *P* = .17].

**Table 3.** Glucocorticoid exposure and SST

Charactersitics	All participants	Pass	Fail	*P*-value
GC exposure history				
Pretransplant GC exposure, *n* (%)	16 (24)	5 (26)	11 (23)	.7
Other current GC exposure, *n* (%)	6 (9)	1 (5)	5 (10)	.66
TDD (mg/pred), median (IQR)	4.7 (4.5–4.9)	4.2 (3.6–4.8)	4.9 (4.7–5)	.002
Cumulative GC exposure (mg pred), median (IQR)	13 050 (7350–29 550)	9220 (5450–21 850)	14 755 (8193–37 275)	.056
Cumulative GC exposure (mg/kg pred), median (IQR)	212 (91–433)	111 (72–284)	289 (115–489)	.03
Cumulative GC exposure (mg pred/kg at time of transplant), median (IQR)	199.54 (102.41–399.71)	141.69 (74.05–274.6)	243.75 (120.59–305.75)	.1023
SST, median (IQR)				
0 min cortisol (nmol/L)	191 (106–272)	303 (238–342)	143 (90–211)	<.001
30 min cortisol (nmol/L)	353 (291–455)	490 (469–531)	325 (272–375)	<.001
Delta cortisol (nmol/L)	152 (116–223)	187 (151–272)	139 (102–195)	.013
0 min ACTH (pg/mL)	14.9 (9.4–20.9)	20 (12.6–23.6)	13.5 (7.3–18.9)	.016
Serum albumin (g/L)	43 (40–46)	45 (41–47)	42 (40–45)	.04
Serum CBG (mg/L)	52.6 (45.9–59.3)	54.4 (50.8–62.5)	51.1 (44.2–59.1)	.17

GC, glucocorticoid; TDD, total daily dose; pred, prednisolone; CBG, cortisol binding globulin.

### Basal cortisol concentration predicts AI risk

**FIGURE 2:**
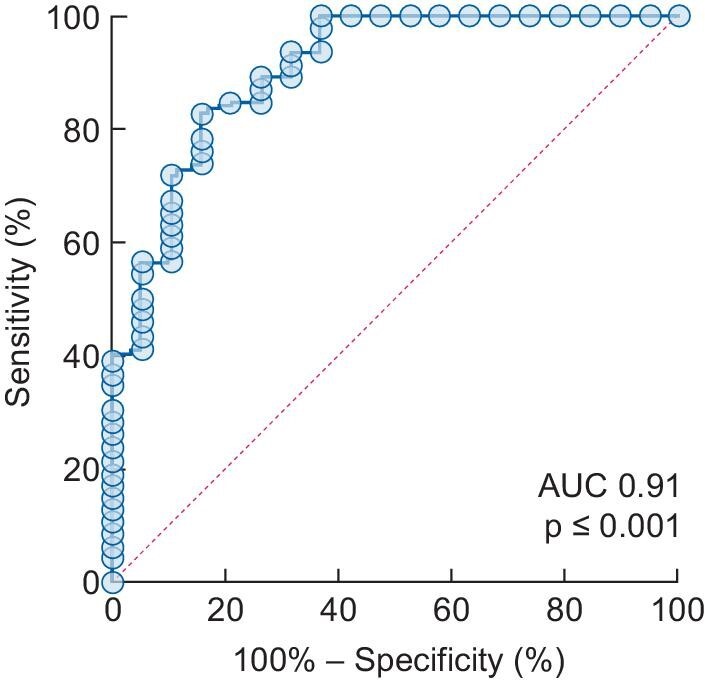
ROC curve demonstrating the performance of a basal serum cortisol concentration to predict the outcome of an SST.

ROC analysis demonstrated the utility of basal serum cortisol in predicting SST outcome (Figure 2). The basal serum is a useful predictor of SST outcome [AUC 0.91 (95% CI 0.82–0.99); *P* < .001]. Basal cortisol <130 nmol/L predicts SST failure with 100% specificity (95% CI 83–100) and 39.13% sensitivity (95% CI 26.39–53.24). Participants with basal serum cortisol <130 nmol/L are therefore unlikely to pass an SST and further testing may not be necessary.

**FIGURE 3:**
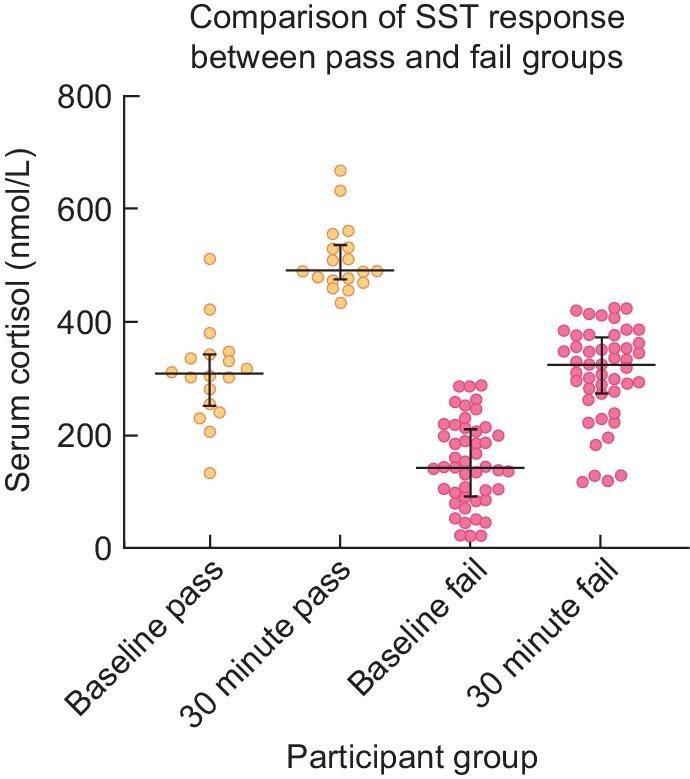
Comparison of morning serum cortisol at baseline and 30 minutes following synacthen injection between participants who passed and failed an SST.

Similarly, basal serum cortisol >288 nmol/L predicts a normal SST response with 100% specificity (95% CI 92–100) and 70% sensitivity (95% CI 56–78) and therefore participants with basal serum cortisol concentration >288 nmol/L may not require SST testing. However, participants with a basal serum cortisol concentration that falls between these threshold values (130–288 nmol/L) would require an SST to exclude the presence of AI. Implementing these threshold values could reduce the number of participants requiring SST by 47%.

### Steroid sick day rules

Only nine patients (13%) were aware of the sick day rules and no patient carried a medical alert bracelet or steroid card.

## DISCUSSION

This is the largest cross-sectional study to report the risk of AI in KTRs receiving low-dose glucocorticoid-based immunosuppression. It is one of the few adult studies to examine the effect of cumulative glucocorticoid exposure on the risk of AI [10, 11] and the only such study to include pre-transplant glucocorticoid exposure. Our data would suggest that AI is common among KTRs receiving glucocorticoid-based immunosuppression, with a prevalence of 72%.

Patients receiving glucocorticoid doses ≥5 mg prednisolone or equivalent for at least 1 month are recognized to be at a high risk of AI [27] and are advised to carry steroid alert cards and follow steroid sick day rules [3, 27]. Our data suggest that patients with prolonged exposure to even lower doses are at risk of AI. AI was seen in participants receiving doses as low as 2.5 mg of prednisolone/day and on alternate-day dosing regimens. AI was seen as early as 14 months following transplantation and normal synacthen responses were seen in participants up to 25 years following transplantation. We would therefore caution against discounting the possibility of AI based on dose or duration of exposure alone.

The prevalence of AI in our cohort is higher than the previously reported rates in KTR cohorts receiving similar daily doses of prednisolone [12, 14, 16] (Table 1). The prevalence of AI in our cohort was also higher than that reported in patients receiving similar daily doses of glucocorticoids for other diagnoses [2, 28]. There are several possible explanations for this, including drug–drug interactions between prednisolone and other immunosuppressants and medication adherence in this transplant population.

The interaction between glucocorticoid medications and other immunosuppressants used in KTRs is complex. Both glucocorticoids and calcineurin inhibitors are substrates for cytochrome P450 3A4 (CYP3A4) enzymes and P-glycoprotein (P-gp) [29, 30]. Glucocorticoids reduce the bioavailability of tacrolimus through enzymatic induction of CYP3A4 and changes the expression of P-gp [31–33]. *In vitro* studies have suggested that tacrolimus may potentiate the effects of glucocorticoids by enhancing nuclear translocation of the glucocorticoid receptor [34]. Pharmacokinetic studies of prednisolone in KTRs have identified high prednisolone tissue exposure, thought to be due to alterations in corticosteroid metabolism via the 11β-hydroxysteroid dehydrogenase enzymes [35]. Therefore, impaired prereceptor metabolism and regulation of glucocorticoid in target tissues may increase prednisolone exposure, thereby adding to the risk of adrenal insufficiency in KTRs.

It is notable that KTRs with lower eGFR were more likely to have AI in our cohort. Studies examining the effects of impaired renal function on the pharmacokinetics of prednisolone have primarily been performed in patients with end-stage renal failure, where uraemia, hypoalbuminaemia and reduced protein binding are hypothesized to increase free prednisolone AUCs [29]. Our cohort displayed a relative preservation of renal function. It is unclear from the literature if mild–moderate renal impairment has an effect on prednisolone pharmacokinetics.

Another possible explanation for the observed high prevalence of AI in the KTR population is adherence to medication in this patient group. The Irish kidney transplant programme operates under the public health system, which offers affordable healthcare to KTRs [36]. It should be noted that the Irish kidney transplant programme has almost 100% recipient follow-up, with failure to attend quarterly transplant clinic appointments a rarity [37, 38].

**FIGURE 4:**
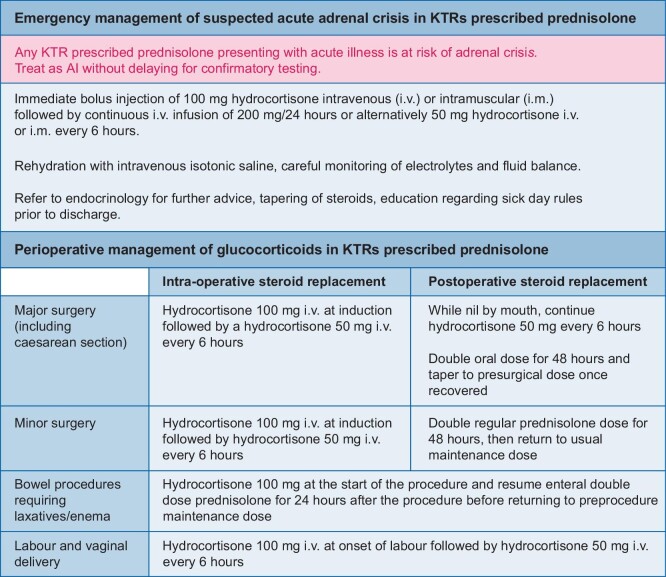
Management of stress-dose glucocorticoids for KTRs prescribed prednisolone.

Any individual with AI is at risk of an adrenal crisis, which can be life-threatening, irrespective of the aetiology. The reported incidence of adrenal crises in patients with known AI is 5.2–8.3 crises/100 patient-years [39, 40], even among patients who have received sick day rules education. Despite this, there are few reported cases of adrenal crises in KTRs in the literature [41, 42]. It is important to consider that adrenal crisis may not be suspected due to a lack of awareness of the risk of AI in this group. Clinicians should have a high clinical suspicion of adrenal crisis in this patient group and treat suspected adrenal crisis without delaying confirmatory testing. KTRs with confirmed AI should be given stress-dose hydrocortisone for an acute illness, diagnostic or therapeutic procedure in the same manner as patients with known AI (Figure 4) [3, 4, 27].

As the majority of KTRs remain on glucocorticoid medication lifelong as part of an immunosuppressive regimen [5], additional glucocorticoid replacement is rarely necessary on a day-to-day basis [2]. However, KTRs with AI receiving prednisolone doses lower than adrenal replacement doses (<3–5 mg/day) [17] may require regular additional glucocorticoid replacement, the need for which must be weighed against the risk of iatrogenic complications of glucocorticoid excess.

Our results also highlight the need for increased focus on patient and healthcare provider education regarding both the risk of AI in KTRs and the prevention of adrenal crisis [43]. Sick day rules education, medic alert bracelets or steroid cards are recommended for all patients with AI [4, 17, 27, 44]. We would suggest that sick day rules education should form part of routine follow-up care in this patient group. Currently neither steroid sick day rules education nor screening for AI are included in the Kidney Disease: Improving Global Outcomes (KDIGO) guidelines for the care of KTRs [6]. A copy of the sick day rules guidance given to the patients enrolled in this study is available as Supplementary data.

Our data would suggest that there is a significant proportion of KTRs with undiagnosed AI. The gold standard for diagnosis of AI is dynamic stimulatory testing [17], which has resource implications when considering screening a large patient population. We suggest that a morning cortisol concentration, measured on a second-generation immunoassay, is a useful alternative to predict the risk of AI in KTRs dividing patients into three groups based on their risk of AI. Patients with a morning cortisol concentration <130 nmol/L should be considered to have AI and should follow sick day rules. KTRs with a morning cortisol concentration of 130–288 nmol/L should be considered a high risk of AI but require an SST to confirm the presence or absence of AI. As glucocorticoid therapy is likely to continue in this group, a safe alternative to testing would be to employ sick day rules and perform SST if glucocorticoid therapy is to be stopped or reduced. A morning cortisol concentration >288 nmol/L virtually excludes AI in this population and therefore SST is not necessary. Repeat interval screening should be considered in this group, as ongoing exposure to glucocorticoids conveys an ongoing risk of AI.

**FIGURE 5:**
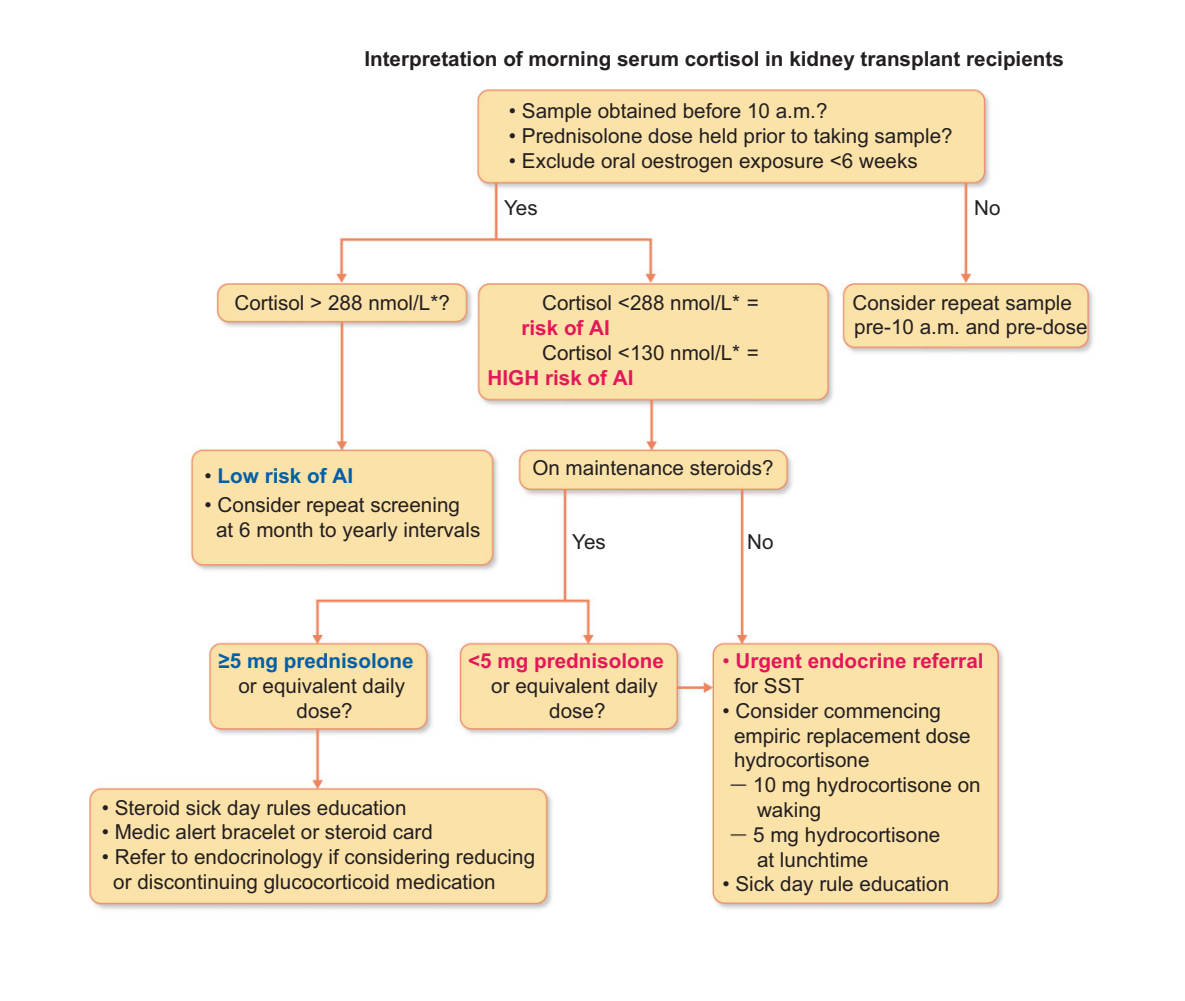
Screening for AI in KTRs using a morning serum cortisol. *Serum cortisol concentration measured using a second-generation immunoassay.

Interval screening with a basal serum cortisol concentration is appropriate for identifying patients (maintained on immunosuppressive glucocorticoid therapy) who are at risk of AI. However, in the event of discontinuation or reduction of glucocorticoid dose below AI replacement dosing (<3–5 mg prednisolone/day), an SST is required to ensure adequate adrenal function. Should these patients have AI they are at risk of adrenal crisis in the absence of adequate glucocorticoid replacement and should be commenced on physiological replacement glucocorticoids such as hydrocortisone 10 mg in the morning and 5 mg in the afternoon. In this case, we would suggest referral to an endocrinologist to ensure that adequate glucocorticoid replacement is prescribed (Figure 5).

In conclusion, AI is common in KTRs receiving low-dose prednisolone as immunosuppressive therapy and is largely undiagnosed. A greater awareness of this risk of AI among both KTRs and clinicians caring for this patient group is key in reducing the potential risk of an adrenal crisis and we would advocate that steroid sick day rules education should be considered as part of routine clinical care for all KTRs receiving glucocorticoid-based immunosuppression. A single morning serum cortisol concentration is a useful screening tool in this patient cohort and may help to identify patients at the highest risk of AI. Finally, we would strongly encourage clinicians to perform an assessment of adrenal function prior to stopping glucocorticoid therapy after a prolonged period of exposure or prior to reducing daily glucocorticoid dosing below an adrenal replacement dose.

## CONFLICT OF INTEREST STATEMENT

None declared. The results presented in this article have not been published previously in whole or part, except in abstract format.

## AUTHORS’ CONTRIBUTIONS

M.T. and J.M.G. were responsible for the conception, design, analysis, interpretation and writing the manuscript. M.T., J.M.G., C.K. and O.E. were responsible for sata collection and study visits. M.T., J.M.G., D.L., P.C., D.D.F., M.D., C.M., C.O.S., M.W.O.R., C.J.T., M.S. provided intellectual content. M.T., J.M.G., C.K., O.E., D.L., P.C., D.D.F., M.D., C.M., C.O.S., M.W.O.R., C.J.T. and M.S. provided critical revision and final approval of the article.

## FUNDING

M.T. is an Irish Clinical Academic Training programme fellow funded by the Health Research Board (HRB) and Wellcome Trust (grant 203930/B/16/Z). J.M.G. receives funding from her fellowship in the Royal College of Surgeons in Ireland/Beacon Hospital Strategic Academic Recruitment programme and the Irish Endocrine Society Clinical Science Award. M.W.O.R. is funded by an HRB Emerging Clinician Scientist Award (ECSA-2020-001).

## DATA AVAILABILITY STATEMENT

The data underlying this article cannot be shared publicly, as participants of this study did not agree for their data to be shared publicly, so supporting data are not available.

## Supplementary Material

gfac044_Supplemental_File
