# Supplementary material for: Adrenal insufficiency is common amongst kidney transplant recipients receiving maintenance prednisolone and can be predicted using morning cortisol
Source: Nephrol Dial Transplant. 2022 May 13;38(1):236–45. doi: 10.1093/ndt/gfac044 (PMC9869856; doi:10.1093/ndt/gfac044)
Supplement: gfac044_Supplemental_File [file gfac044_supplemental_file.docx]

| **Patient Information**  **Steroid Medication**  **(Prednisolone)** |
| --- |
| You are or you could be deficient in a hormone called Cortisol.  Cortisol is a steroid produced by the adrenal gland under control from the pituitary gland. It plays a vital role in health and disease including when trying to cope with physical stress such as an infection, acute illness, trauma and surgical procedures.  Because you take regular prednisolone (which is a steroid tablet) you have enough steroid hormone for every-day life. However, in times of acute illness or surgery you may require more steroid hormone to overcome this stress.  It is very important to take your prednisolone as prescribed and do **NOT** stop taking your prednisolone without medical advice. |
| **Acute illness**  Should you suffer an illness **you should immediately increase your dose of prednisolone to 10mg** while you are unwell. Only go back to taking your normal dose when you have recovered. For example, if you suffer from a kidney infection, chest infection, viral illness or if you need to take antibiotics, take to the bed or take time off work you should increase your prednisolone dose as above. |
| If you are about to undergo a surgical procedure, you should inform the relevant doctors and show them this leaflet. They will increase your prednisolone as appropriate and usually they will give it to you initially by injection. |
| If you are not able to take your prednisolone due to vomiting, try taking it again.  If you are still unable to take your tablets you will require a hydrocortisone injection to be given without delay by your GP or in your local accident and emergency department. In some cases, a family member can be shown how to give this injection. |
| You should also wear a medical alert bracelet or necklace (obtainable from pharmacies) so that in an emergency situation it will alert others that you are cortisol deficient**.** |
| **Going on holidays**  When going on holiday especially abroad it is important that you obtain from your GP an up to date letter giving details of your medical condition and your fitness to travel.  It is very important that you bring an adequate supply of prednisolone tablets to allow for any increase in your dose should you become unwell. |
| **If you are not sure what to do, double your dose of steroid**  **and seek medical advice.** |
